# Pressure measurement characteristics of a micro‐transducer and balloon catheters

**DOI:** 10.14814/phy2.14831

**Published:** 2021-05-02

**Authors:** William MacAskill, Ben Hoffman, Michael A. Johnson, Graham R. Sharpe, Dean E. Mills

**Affiliations:** ^1^ Respiratory and Exercise Physiology Research Group School of Health and Wellbeing University of Southern Queensland Ipswich Australia; ^2^ Centre for Health Research Institute for Resilient Regions University of Southern Queensland Ipswich Australia; ^3^ School of Human Movement and Nutrition Sciences The University of Queensland Brisbane Australia; ^4^ Exercise and Health Research Group, Sport, Health and Performance Enhancement (SHAPE) Research Centre School of Science and Technology Nottingham Trent University Nottingham United Kingdom

**Keywords:** balloon catheter, Esophageal catheter, micro‐transducer catheter, respiratory pressures

## Abstract

Respiratory pressure responses to cervical magnetic stimulation are important measurements in monitoring the mechanical function of the respiratory muscles. Pressures can be measured using balloon catheters or a catheter containing integrated micro‐transducers. However, no research has provided a comprehensive analysis of their pressure measurement characteristics. Accordingly, the aim of this study was to provide a comparative analysis of these characteristics in two separate experiments: (1) *in vitro* with a reference pressure transducer following a controlled pressurization; and (2) *in vivo* following cervical magnetic stimulations. *In vitro* the micro‐transducer catheter recorded pressure amplitudes and areas which were in closer agreement to the reference pressure transducer than the balloon catheter. *In vivo* there was a main effect for stimulation power and catheter for esophageal (P_es_), gastric (P_ga_), and transdiaphragmatic (P_di_) pressure amplitudes (*p* < 0.001) with the micro‐transducer catheter recording larger pressure amplitudes. There was a main effect of stimulation power (*p* < 0.001) and no main effect of catheter for esophageal (*p* = 0.481), gastric (*p* = 0.923), and transdiaphragmatic (*p* = 0.964) pressure areas. At 100% stimulator power agreement between catheters for P_di_ amplitude (bias =6.9 cmH_2_O and LOA −0.61 to 14.27 cmH_2_O) and pressure areas (bias = −0.05 cmH_2_O·s and LOA −1.22 to 1.11 cmH_2_O·s) were assessed. At 100% stimulator power, and compared to the balloon catheters, the micro‐transducer catheter displayed a shorter 10–90% rise time, contraction time, latency, and half‐relaxation time, alongside greater maximal rates of change in pressure for esophageal, gastric, and transdiaphragmatic pressure amplitudes (*p* < 0.05). These results suggest that caution is warranted if comparing pressure amplitude results utilizing different catheter systems, or if micro‐transducers are used in clinical settings while applying balloon catheter‐derived normative values. However, pressure areas could be used as an alternative point of comparison between catheter systems.

## Graphical abstract

The aim of this study was to provide a comparative analysis of micro‐transducer and balloon catheter pressure characteristics: (1) in vitro with a reference pressure transducer; and (2) in vivo following cervical magnetic stimulations. In vivo there was a main effect for catheter for esophageal, gastric, and transdiaphragmatic pressure amplitudes and no main effect for pressure areas, indicating that pressure areas could be used as a point of comparison between catheter systems. Caution is warranted if comparing pressure amplitude results from different catheter systems or if micro‐transducers are used in clinical settings with balloon catheter‐derived normative values.

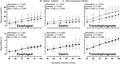

## 1 INTRODUCTION

Respiratory pressure responses to nerve stimulation are important measurements in monitoring the mechanical function of the respiratory muscles (American Thoracic Society, 2003, Laveneziana et al., 2019, Macklem, 2004, Romer & Polkey, 2008). As measurements of pleural and abdominal pressures are invasive, they are typically estimated using surrogate measures of esophageal (P_es_) and gastric (P_ga_) pressures, respectively (Benditt, 2005, Laveneziana et al., 2019). Traditionally, these measurements are collected with balloon catheters (Baydur et al., 1982; Milic‐Emili et al., 1964), but variations in catheter design, manual inflation of the balloon with either air or fluid, and catheter placement can lead to under or overestimation of pressure (Mead et al., 1955; Milic‐Emili et al., 1964; Mojoli et al., 2015; Petit & Milic‐Emili, 1958; Walterspacher et al., 2014).

There are a variety of commercially available balloon catheter designs and each requires a different quantity of air for optimum performance, and under and over inflation of balloons can produce invalid estimations of pressure (Milic‐Emili et al., 1964; Mojoli et al., 2015; Walterspacher et al., 2014). The perimeter and length of a balloon, along with its elastance, can also affect measurement accuracy (Mead et al., 1955; Mojoli et al., 2015; Petit & Milic‐Emili, 1958). Pressures are also affected by the location of the balloon within the body and are therefore dependent on placement technique (Mead & Whittenberger, 1953; Petit & Milic‐Emili, 1958). The proximal end of a balloon catheter is attached via plastic tubing to a pressure transducer located outside the body. Increasing the tubing length between the balloon and the transducer leads to reduced flow within the tubing (i.e., Poiseuille's Law), which may compromise dynamic response characteristics in balloon catheter systems (Cross et al., 2016; Mead et al., 1955; Mojoli et al., 2015; Walterspacher et al., 2014). Furthermore, balloon elasticity may change over time due to repeated sterilization and re‐use. These issues may explain the limited uptake of balloon catheters in clinical settings (Mauri et al., 2016; Mojoli et al., 2015) despite their many medical applications (Akoumianaki et al., 2014; Mauri et al., 2016).

The primary alternative to a balloon catheter is a catheter containing one or two integrated micro‐transducers (Beardsmore et al., 1982; Evans et al., 1993; Gilbert et al., 1979). Since micro‐transducer catheters do not utilize a balloon or require tubing to connect to an external transducer, they may overcome some of the limitations associated with traditional balloon catheters. However, despite these benefits, micro‐transducer measurements of P_es_ are more susceptible to mucus adhesion and contact with the esophageal wall, which reduces the surface area and therefore the spread of Van der Waals forces (Peters et al., 1998). Unpredictable shifts in baseline P_es_ have also been reported and are partly attributed to the micro‐transducers susceptibility to differences in pressures across the esophagus (Beardsmore et al., 1982), to regional artifacts (Panizza & Finucane, 1992) and baseline pressure drift in the device over time (1999). Recently, Augusto et al. reported no clinically relevant drift following 1 h of submersion with a Gaeltech micro‐transducer catheter. Micro‐transducer measurements of P_ga_ may be also affected by immersion in gastric fluids (Stell et al., 1999).

Despite the potential benefits of the micro‐transducer catheter, only a limited number of studies have compared their pressure responses with those of a balloon catheter, and the results remain controversial. Poor agreement has been reported for absolute P_es_ and P_ga_ (Augusto et al., 2017; Beardsmore et al., 1982; Peters et al., 1998; Stell et al., 1999), whereas both good (Peters et al., 1998; Stell et al., 1999) and poor (Augusto et al., 2017; Beardsmore et al., 1982) agreement has been reported for relative P_es_ and P_ga_ (i.e., amplitude relative to baseline). Moreover, ambiguous evidence is provided by other studies that describe micro‐transducer and balloon catheters as “measuring pressures similarly” (Evans et al., 1993) and as “providing comparable measurements of absolute P_es_ and P_ga_” (Gilbert et al., 1979). As such, it is not clear how comparable the two devices are and which device measures pressure more accurately.

Analysis of magnetic or electrical cervical stimulation is important for the comprehensive assessment of the mechanical and neural properties of the respiratory muscles (Laghi et al., 1996; Man et al., 2004; Similowski et al., 1989, 1991, 1996, 1998; Taylor et al., 2006). Thus, understanding the accuracy and comparability of the two devices in measuring these responses is important for the correct interpretation of these measurements. While previous studies have evaluated the differences in pressures between balloon and micro‐transducer catheters (Augusto et al., 2017; Beardsmore et al., 1982; Panizza & Finucane, 1992; Stell et al., 1999), none have provided a comprehensive analysis of their pressure measurement characteristics following electric or magnetic stimulations. Accordingly, this study provides a thorough assessment of a range of characteristics for P_es_, P_ga_, and transdiaphragmatic pressure (P_di_) in response to controlled pressurizations *in vitro* and to cervical magnetic stimulation *in vivo*.

## 2 METHODS

### 2.1 Experimental overview

This study comprised two separate experiments to evaluate the pressure measurement characteristics of a micro‐transducer catheter and balloon catheters. Experiment 1 evaluated, *in vitro*, the pressure amplitudes and areas of both catheter types following a controlled pressurization, with their responses compared to a reference pressure. Experiment 1 was also used to identify whether differences in catheter responses are present after removal of physiological factors such as mucus adhesion and immersion in gastric fluids. Experiment 2 evaluated, *in vivo*, the characteristics of both catheter types in human participants following cervical magnetic stimulation. The study was approved by the University of Southern Queensland's Ethics Committee and all procedures conformed to the standards set by the Declaration of Helsinki.

#### 2.1.1 Experiment 1—in vitro

##### Protocols

The micro‐transducer catheter and a single balloon catheter were positioned in a sealed pressurized polyvinylchloride chamber (length =25 cm; radius =1 cm) alongside a reference pressure transducer (piezo‐resistive pressure transmitter MRB20; Bestech, Brisbane, Australia). The reference pressure was the standard against which pressures recorded by the micro‐transducer and balloon catheters were compared (measurement range =500 cmH_2_O; frequency response =1 kHz). The reference pressure transducer was calibrated at room temperature using a water manometer with a 1 m water column. The balloon catheter was inflated with 1 mL of air from a glass syringe, and both catheter types were then calibrated within the chamber at 100 cmH_2_O as measured by the reference pressure transducer. The catheters were then exposed to chamber pressures of 25, 50, 75, and 100 cmH_2_O (*n* = 100 for each) with a constant pressurization time of 0.2 s. For experiment 1, the same micro‐transducer catheter and a single balloon catheter were used, and all measurements were taken on the same day.

The micro‐transducer catheter and balloon catheter were secured on a mounting board with the micro‐transducers aligned to the centers of the balloons. This assembly and the reference pressure transducer were placed inside the airtight chamber which was pressurized using a gas supply (79% N_2_, 16% O_2_, and 5% CO_2_; BOC, North Ryde, Australia). The cylinder was fitted with a Type 10 valve (flow coefficient =0.4; BOC, North Ryde, Australia) leading to a regulator (6000 Argon Gas Regulator; BOC) with an upstream pressure of 2900 PSI. Maximum chamber pressures were adjusted via the regulator to obtain maximum pressure at the end of a 0.2 s pressurization time. Pressurization was automated using the Powerlab 16/35 to control a two‐way normally open isolation valve (NR3‐2–12; VFV, Mitcham, Australia). When the gas flow was switched off by the isolation valve, depressurization was complete within 150 – 250 ms.

#### 2.1.2 Experiment 2—in vivo

##### Participants

Healthy young male (*n* = 4) and female (*n* = 4) participants (age =29 ± 3 years; height =173 ± 11 cm; body mass =84.7 ± 9.6 kg) with normal pulmonary function (forced vital capacity =98 ± 9% predicted; forced expiratory volume in 1 s = 95 ± 9% predicted) provided written informed consent to participate in this study. Exclusion criteria included current cigarette smokers, a history or current symptoms of cardiopulmonary disease, and a body mass index of <18.5 or >30 kg/m^2^.

##### Experimental design

Each participant visited the laboratory on two occasions, at a similar time of day, separated by a minimum of 24 h and a maximum of 7 days. Before each visit, participants abstained from food for 4 h, caffeine for 12 h, and exercise for 48 h. During visit 1, anthropometric measures and pulmonary function were assessed using a spirometer (Vmax® Encore PFT system; Vyaire Medical, Chicago, USA) according to published guidelines (Miller et al., 2005). Participants were instrumented with a micro‐transducer catheter to evaluate P_es_, P_ga_, and P_di_ responses to cervical magnetic stimulation. The micro‐transducer catheter was then removed, and participants were instrumented with esophageal and gastric balloon catheters and pressure responses to cervical magnetic stimulation were re‐evaluated. During visit 2, the order of catheter placement was reversed. The duration between removal of catheter(s) and instrumentation of the next catheter(s) was ~10 min.

##### Respiratory pressure catheters

The micro‐transducer catheter (Gaeltech) housed two pressure transducers (~5 × 2 mm), separated by 22.8 cm, which were constructed using half‐bridge thin‐film resistive strain gauge sensors coated with a silicone elastomer with frequency responses of 10–20 kHz. The catheter comprised a 100 cm silicon shaft (2.7 mm diameter) that also contained nine silver electrodes spaced 1 mm apart (electromyography data not reported here) and the pressure transducers were positioned proximally and distally to the electrodes. Prior to instrumentation *in vivo* the catheter was soaked for 1 h as per manufacturer's instructions to reduce baseline drift. The micro‐transducer catheter was then placed inside a small section of airtight plastic tubing and calibrated by injecting or withdrawing air, via a three‐way open valve connected to a glass syringe and a handheld respiratory pressure meter (Micro RPM; Vyaire Medical, Chicago, USA). P_es_ was calibrated to −100 cmH_2_O and P_ga_ to +100 cmH_2_O. The external transducers of the balloon catheters were connected, via a 3‐way open valve, directly to the respiratory pressure meter and glass syringe. These transducers were calibrated between −27 cmH_2_O and +100 cmH_2_O by injecting and withdrawing air. The two balloon catheters consisted of a thin‐walled (~0.6 mm) polytetrafluoroethylene balloon (9.5 cm in length) sealed over an 86‐cm‐long polyethylene catheter (Adult esophageal balloon catheter; Cooper Surgical). These were connected to external pressure transducers with maximum frequency responses of 300 Hz and a pressure range of −27 to 407 cmH_2_O (SP844 Pressure Transducer; MEMSCAP). P_di_ was calculated automatically using LabChart Pro software (AD Instruments, Bella Vista, Australia) by subtracting P_es_ from P_ga_.

##### Catheter placement

Catheter placement was preceded by intranasal administration of 1 mL of anesthetic lidocaine hydrochloride gel (Instillagel; MD Solutions Australasia). The positioning of the micro‐transducer catheter was achieved as previously described (Luo et al., 2001). The catheter was passed peri‐nasally into the stomach until a positive deflection in P_ga_ and a negative deflection in P_es_ were observed during repeated sniffs. The catheter was then repositioned based on the strength of the crural diaphragm EMG simultaneously from different pairs of electrodes and was then secured in place. An occlusion test was then performed to confirm the catheter's location in the esophagus (Baydur et al., 1982). As esophageal diaphragm EMG is sensitive to differences in positioning (Luo et al., 2000), the micro‐transducer was positioned first to ensure the collection of quality EMG data. Subsequently, the deflated balloon catheters were inserted through the same nostril used for the micro‐transducer catheter. The centers of the respective balloons were positioned at the same distance from the nares as the micro‐transducers. The esophageal and gastric balloons were inflated with 1 and 2 mL of air, respectively. P_es_ and P_ga_ deflections were then observed during repeated sniffs to check positioning, before being further assessed by an occlusion test. If required, the location of the balloon catheters was then altered to ensure accurate P_es_ and P_ga_ measurements. The position of the catheters, relative to the nares, was identical during visits 1 and 2. This process allowed for the optimization of P_es_, P_ga_, and EMG signals.

##### Cervical magnetic stimulation

After an initial 20‐min seated rest period to minimize post‐activation potentiation (Wragg et al., 1994), cervical magnetic stimulation was performed using a 90‐mm circular coil attached to a magnetic stimulator (200^2^; Magstim, Whitland, United Kingdom). Participants wore a nose‐clip and were seated in a chair with their neck flexed. Stimulations were performed with the glottis closed at functional residual capacity, which was inferred from visual feedback of P_es_ (i.e., an elevated plateau at the end of a tidal breath). The optimal stimulation site was determined by performing multiple stimulations at submaximal intensity (50% stimulator power) along C5‐C7 until the maximal P_di_, and thus the optimal stimulation site, was determined. This site was marked with indelible ink and used for all subsequent stimulations. P_es_, P_ga_, and P_di_ amplitudes were not different between visits, indicating that all stimulations were delivered with the same thoracoabdominal configuration. Pressure systems were compared at intensities of 50, 60, 70, 80, 85, 90, 95, and 100% of stimulator power output, with a minimum of three stimulations recorded at each intensity. Additional stimulations were performed when P_es_ or P_ga_ values at end‐expiration were not at a stable baseline value. A 30 s pause was maintained between stimulations to prevent twitch‐on‐twitch potentiation (Guenette et al., 2010; Polkey et al., 1995; Taylor & Romer, 2009; Welch et al., ,^2017^, ^2018^).

### 2.2 Pressure capture and response analyses

**FIGURE 1:**
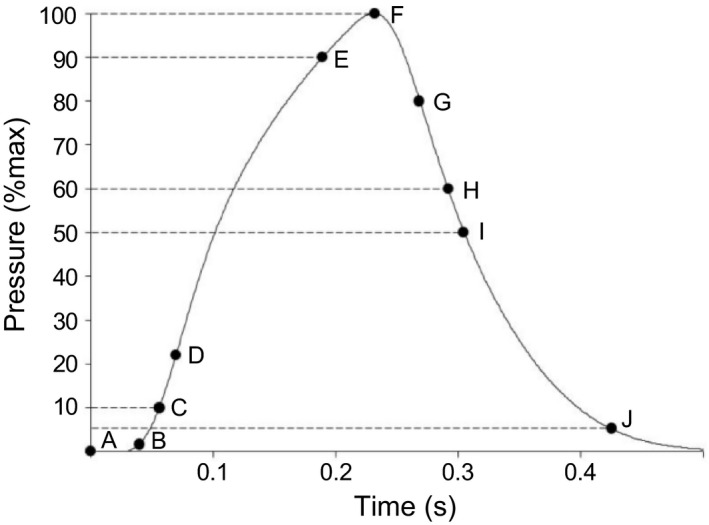
Pressure response analysis. (A) stimulation event; (B) pressure 5% above baseline; (A‐B) latency; (C‐E) 10–90% rise time; (D) point of the maximal rate of pressure development calculated as derivative at D divided by pressure amplitude at (F; G) point of the maximal relaxation rate calculated as derivative at G divided by pressure amplitude at F; (F) peak pressure; (A‐F) time to peak pressure; (B‐F) contraction time; (F‐I) half‐relaxation time; (H‐J) time constant calculated from 60–5% pressure amplitude

Pressures were amplified with a Quad Bridge Amplifier (FE224; ADInstruments, Bella Vista, Australia) and all data were sampled continuously at 10 kHz using a Powerlab 16/35 and recorded using LabChart v8.1.2 software (ADInstruments). Low pass filters were set at 10 Hz for the balloon catheter pressure transducers and 1 kHz for the micro‐transducer catheter and the reference pressure transducer. In experiment 1, pressure amplitudes and areas were analyzed. In experiment 2, pressure amplitude, percentage of maximum amplitude, latency, contraction time, pressure area, 10–90% rise time, half‐relaxation time, time constant, maximal rate of pressure development (MRPD), maximal relaxation rate (MRR), and time to peak pressure using customized macroinstructions (LabChart v8.1.2 software; ADInstruments) (Figure 1).

Pressure amplitude was calculated as the difference between baseline and peak pressure. Response onset was defined as the point at which pressure deviated 5% from baseline. Offset was defined as the point at which pressure returned to ±5% of baseline. Latency was defined as the time difference between magnetic stimulation and response onset (Experiment 2) or the time difference between valve opening and response onset (Experiment 1). Contraction time was defined as the duration between response onset and 100% of peak pressure. Pressure area was calculated using the integration between response onset and offset. The 10–90% rise time was defined as the elapsed time between 10% and 90% of peak pressure. Half‐relaxation time was defined as the elapsed time between 100% and 50% of peak pressure. The time constant was calculated between 60% and 10% of pressure amplitude. Time to peak pressure was defined as latency plus contraction time. MRPD and MRR were calculated based on equations [1] and [2] from previous work (Similowski et al., 1991).

(1) $$MRPD=\max\left|\frac{\mathrm{dP}}{\mathrm{dt}}\right|÷A$$

(2) $$MRR=\max\left|\frac{\mathrm{dP}}{\mathrm{dt}}\right|÷A$$

Where *dP*/*dt* is the rate of change in pressure and *A* is the amplitude of the pressure response.

#### 2.2.1 Statistical analyses

Statistical analyses were performed using SPSS for Windows (IBM). An initial power calculation was performed on the basis of the P_di_ amplitudes for the balloon catheters and micro‐transducer catheter following cervical magnetic stimulation at 100% of stimulator power output. Power analysis indicated that a sample size of 8 would be required to detect differences in P_di_ amplitudes between catheters (alpha =0.05 and power =0.8). Normality was assessed using a Shapiro–Wilk test. Supramaximality was determined by identifying a plateau in mean twitch P_di_ at increasing stimulation power using a one‐way repeated measures ANOVA followed by pairwise comparisons (Guenette et al., 2010).

Between‐visit and between‐catheter pressure measurement characteristics at 100% of maximum stimulator output in response to cervical magnetic stimulation were analyzed using a paired sample t‐tests or Wilcoxon signed ranks test for parametric and nonparametric data, respectively. Between‐catheter differences for pressure amplitudes and areas at increasing stimulation intensities were analyzed using a two‐way repeated measures ANOVA to determine the effects of stimulation “intensity” (50, 60, 70, 80, 85, 90, 95, and 100% of maximum stimulation output) and “catheter” (micro‐transducer vs. balloon catheter). Significant intensity ×catheter interaction effects were followed by planned pairwise comparisons between catheters using the Bonferroni method.

The agreement, relationship, and reliability characteristics for pressure amplitudes and areas between the micro‐transducer catheter and balloon catheters were determined from data collected from all chamber pressures (Experiment 1—*in vitro*) or stimulation intensities (Experiment 2—*in vivo*). Bland–Altman analysis was used to evaluate the agreement between balloon and micro‐transducer catheter pressure measurements (Giavarina, 2015). Bias was defined as the micro‐transducer catheter measurement minus the balloon catheter measurement (experiment 1, *in vivo*), or as the reference transducer measurement minus the catheter measurement (experiment 2, *in vitro*). Limits of agreement (LOA) were calculated as the mean difference (bias) ±1.96 SD. Pearson's product moment correlation coefficient was used to examine the relationship between catheters. Within‐day reliability was assessed using coefficients of variation (CV) with the method error of the measurement (i.e., standard deviation divided by the mean). Between‐day reliability was assessed by using CV and the intraclass correlation coefficient (ICC(2,k)). Statistical significance was set at *p* < 0.05. Results are presented as means ±SD unless stated otherwise.

## 3 RESULTS

### 3.1 Experiment 1—in vitro

**FIGURE 2:**
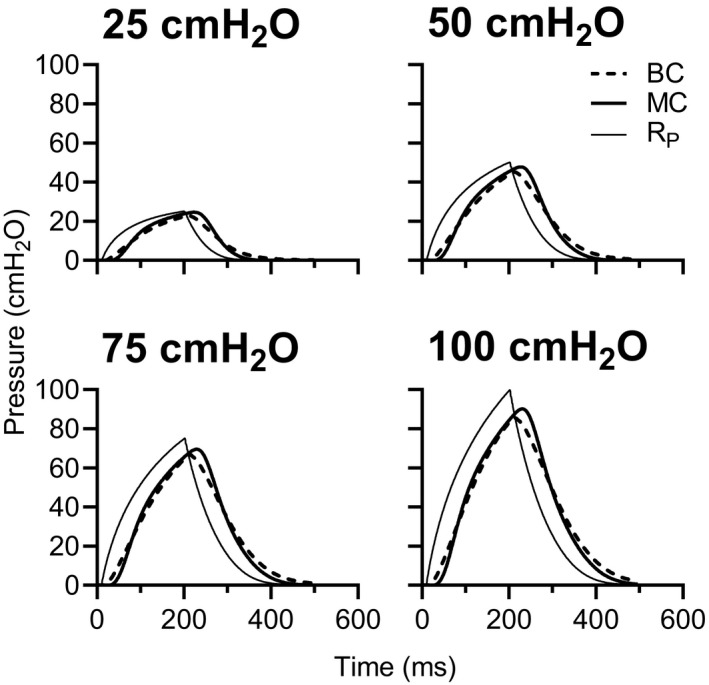
Experiment 2—in vivo: Bland–Altman plots of esophageal, gastric, and transdiaphragmatic pressure amplitudes (top panels) and areas (bottom panels) between balloon catheters (BC) and micro‐transducer catheter (MC) following cervical magnetic stimulation at increasing stimulation intensities. Bias is represented by the solid line and the limits of agreement by the dotted lines (± 1.96 SD). Each participant has one datapoint per stimulation power and each datapoint was calculated as the mean value from visits 1 and 2

Ensemble averaged pressure responses to increasing chamber pressurizations for the micro‐transducer catheter, balloon catheter, and reference transducer are shown in Figure 2. Table 1 shows the measurement characteristics and agreement for pressure amplitudes and areas between the micro‐transducer catheter and balloon catheter and at increasing chamber pressures of 25, 50, 75, and 100 cmH_2_O with a constant pressurization time of 0.2 s. Pressure amplitudes were higher for the micro‐transducer catheter compared to the balloon catheter at all chamber pressures. Pressure areas for the micro‐transducer catheter were slightly higher than for the balloon catheter, with some exceeding that of the reference pressure at chamber pressures of 25 and 50 cmH_2_O, respectively (Table 1). Despite this, micro‐transducer catheter pressure amplitudes and areas were closer to reference values than the balloon catheters with the largest differences between the catheters occurring at the lowest chamber pressure (25 cmH_2_O; Table 1).

**TABLE 1:** Experiment 1—*in vitro*: Measurement characteristics and agreement for pressure amplitudes and areas between the balloon catheter (BC) and micro‐transducer catheter (MC) at increasing chamber pressures of 25, 50, 75, and 100 cmH_2_O with a constant pressurization time of 0.2 s. Bias values were calculated as catheter pressure subtracted from reference pressure. Values are mean ±SD calculated from 100 responses to each chamber pressure

	25 cmH_2_O		50 cmH_2_O		75 cmH_2_O		100 cmH_2_O	
	BC	MC	BC	MC	BC	MC	BC	MC
Amplitude (cmH_2_O)	22.8 ± 0.1	24.7 ± 0.1	44.9 ± 0.1	47.7 ± 0.1	66.2 ± 0.1	69.3 ± 0.1	84.8 ± 0.1	89.7 ± 0.1
Amplitude (%R_P_)	91 ± 0	99 ± 0	90 ± 0	95 ± 0	89 ± 0	93 ± 0	86 ± 0	90 ± 0
Amplitude Bias (cmH_2_O)	2.2	0.4	5.0	2.3	8.6	5.5	14.4	9.6
Amplitude LOA (cmH_2_O)	2.2 to 2.3	0.3 to 0.5	5.0 to 5.1	2.2 to 2.4	8.5 to 8.7	5.4 to 5.6	13.8 to 15.0	8.9 to 10.2
Area (cmH_2_O·s)	4.17 ± 0.02	4.39 ± 0.02	8.59 ± 0.02	8.83 ± 0.02	13.2 ± 0.03	13.4 ± 0.03	17.8 ± 0.03	18.0 ± 0.04
Area (%R_P_)	97 ± 0	102 ± 0	98 ± 0	101 ± 0	98 ± 0	99 ± 0	97 ± 0	98 ± 0
Area Bias (cmH_2_O·s)	0.12	−0.10	0.17	−0.08	0.24	0.08	−0.51	−0.36
Area LOA (cmH_2_O·s)	0.11 to 0.13	−0.12 to −0.08	0.16 to 0.18	−0.11 to −0.05	0.22 to 0.26	0.04 to 0.11	−0.52 to −0.49	−0.39 to −0.33

Abbreviations: R_P_, reference pressure; LOA, limits of agreement (bias ±1.96 SD).

For pressure amplitudes and areas, agreement with the reference pressure transducer was closer (reflected by a lower bias) for the micro‐transducer catheter than the balloon catheter (Table 1). Significant correlations between the catheters for pressure amplitude were present at chamber pressures of 25 (*r* = 0.84), 50 (*r* = 0.78), 75 (*r* = 0.91), and 100 (*r* = 0.91) cmH_2_O (*p* < 0.001). Similarly, correlations between the catheters for pressure area were also present at chamber pressures of 25 (*r* = 0.77), 50 (r = 0.79), 75 (*r* = 0.84), and 100 (*r* = 0.90) cmH_2_O (*p* < 0.001). Within‐day reliability was high for both micro‐transducer and balloon catheters for pressure amplitudes (micro‐transducer vs. balloon catheters): 0.25 (CI 0.22 to 0.27) vs. 0.22 (CI 0.20 to 0.24%) and areas 0.29 (CI 0.27 to 0.31) vs. 0.25 (CI 0.24 to 0.27) %.

### 3.2 Experiment 2—in vivo

**FIGURE 3:**
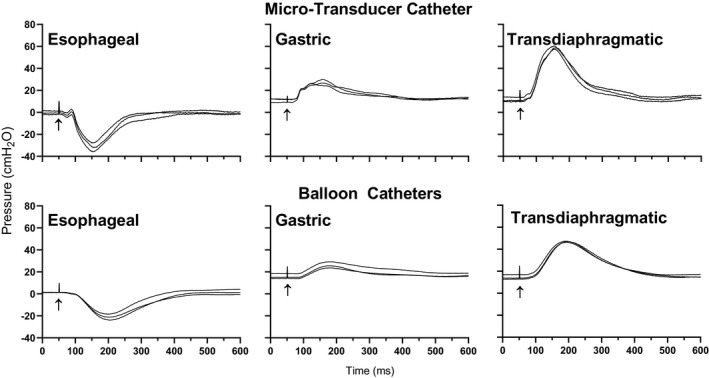
Experiment 1—in vitro: Ensemble average waveforms (each from 100 waves) from the micro‐transducer catheter (MC), balloon catheter (BC), and reference (R_P_) pressures in response to chamber pressures of 25, 50, 75, and 100 cmH_2_O with a constant pressurization time of 0.2 s

Representative pressure responses to cervical magnetic stimulation at 100% of stimulator power output for the balloon catheters and micro‐transducer catheter are shown in Figure 3. There were no between‐visit differences for all pressure measurement characteristics for the micro‐transducer (*p* = 0.055) and balloon catheters (*p* = 0.314). Therefore, data from visits 1 and 2 were pooled. Supramaximality was achieved from 80% (*p* > 0.055) and 90% (*p* > 0.105) stimulator power output for the balloon and micro‐transducer catheters.

Table 2 shows the P_es_, P_ga_, and P_di_ pressure measurement characteristics for the balloon catheters and micro‐transducer catheter following cervical magnetic stimulation at 100% of stimulator power output. Compared to the balloon catheters, the micro‐transducer catheter displayed shorter 10–90% rise times, contraction times, latencies and half‐relaxation times, and greater maximal rates of changes in pressure (MRPD and MRR) and pressure amplitudes (*p* < 0.05). When pressure amplitudes were normalized to the percentage of maximum, there was no difference between catheters, nor were there any differences between catheters for pressure area. P_ga_ and, subsequently, P_di_ were higher (*p* < 0.05) at end‐expiration for the micro‐transducer catheter than the balloon catheters.

**TABLE 2:** Experiment 2—*in vivo*: Esophageal pressure (P_es_), gastric pressure (P_ga_), and transdiaphragmatic pressure (P_di_) measurement characteristics for balloon catheters (BC) and micro‐transducer catheter (MC) following cervical magnetic stimulation at 100% of stimulator power output. Data are mean ±SD and pooled from visits 1 and 2

	P_es_		P_ga_		P_di_	
	BC	MC	BC	MC	BC	MC
Amplitude (cmH_2_O)	15.8 ± 4.1*	20.5 ± 6.4	9.0 ± 3.1*	13.1 ± 4.2	24.2 ± 5.0*	32.1 ± 8.3
Amplitude (%max)	89 ± 9	87 ± 12	78 ± 16	74 ± 19	94 ± 4	92 ± 5
Area (cmH_2_O·s)	2.4 ± 0.7	2.3 ± 0.7	2.9 ± 1.3	2.5 ± 1.6	4.5 ± 0.9	4.3 ± 1.2
10–90% Rise time (ms)	66 ± 9*	43 ± 8	78 ± 21*	38 ± 18	69 ± 8*	47 ± 8
Time to peak pressure (ms)	97 ± 13*	66 ± 12	121 ± 36*	58 ± 28	146 ± 13*	95 ± 12
Latency (ms)	49 ± 5*	33 ± 6	39 ± 3*	27 ± 7	42 ± 3*	27 ± 3
Half‐relaxation (ms)	89 ± 12*	60 ± 12	132 ± 67*	82 ± 58	108 ± 14*	70 ± 7
Time constant (ms)	70 ± 30	54 ± 24	197 ± 182	125 ± 135	106 ± 13	98 ± 39
MRPD (%gain/10 ms)	12.8 ± 2.1*	18.4 ± 1.7	13.6 ± 2.3*	18.7 ± 2.9	12.6 ± 1.4*	17.3 ± 1.8
MRR (%loss/10 ms)	8.1 ± 2.3*	10.4 ± 2.5	5.9 ± 3.2*	8.2 ± 3.2	5.6 ± 0.7*	8.9 ± 2.0
Pressure at end‐expiration (cmH_2_O)	−1.4 ± 2.1	0.8 ± 2.5	13.5 ± 5.1*	10.6 ± 2.2	16.0 ± 3.5*	9.7 ± 3.0

Note

Significantly different from micro‐transducer catheter (*p < 0.05).

Abbreviations: MRPD, maximum rate of pressure development; MRR, maximum rate of relaxation.

**FIGURE 4:**
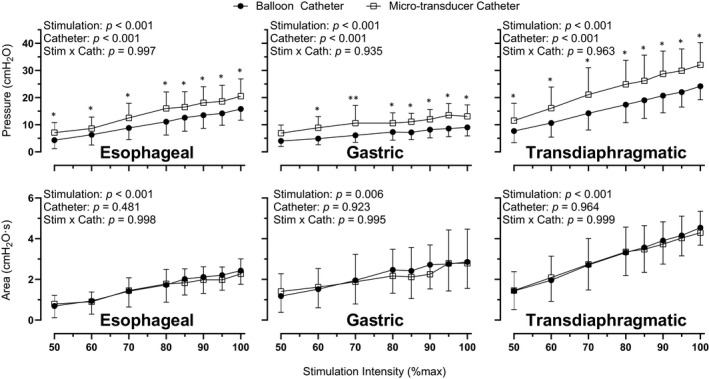
Experiment 2—*in vivo*: Representative esophageal, gastric, and transdiaphragmatic pressure characteristics for the balloon catheters and micro‐transducer catheter following cervical magnetic stimulation at 100% of stimulator power output. Three repeated twitches from one participant are shown superimposed. Stimulation artifacts are marked with an arrow (↑)

P_es_, P_ga_, and P_di_ amplitudes and areas from the micro‐transducer and balloon catheters in response to increasing stimulation intensities are shown in Figure 4. Both catheters responded linearly to increasing stimulation intensities. For P_es_, P_ga_, and P_di_ amplitude, there were main effects of stimulation intensity (*p* < 0.001) and catheter (*p* < 0.001). That is, pressure amplitudes increase with stimulation intensity and are higher for the micro‐transducer catheter. No intensity ×catheter interaction effects (*p* > 0.935) were observed. For P_es_, P_ga_, and P_di_ pressure areas, there was a main effect of stimulation intensity (*p* < 0.001) with pressure area increasing with stimulation intensity. There were no main effects of catheter (*p* = 0.481) or stimulation intensity ×catheter interaction effects (*p* > 0.995).

**FIGURE 5:**
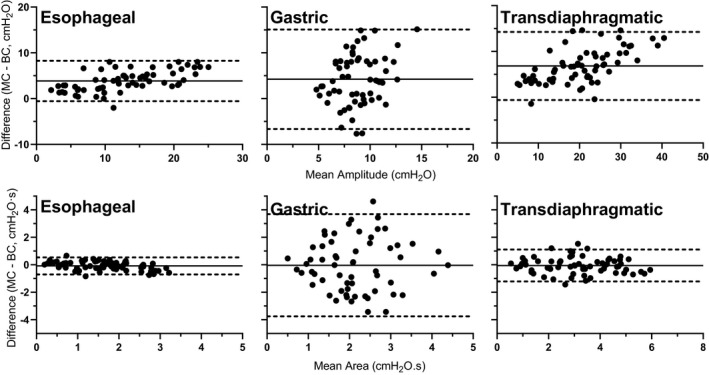
Experiment 2—*in vivo*: Esophageal, gastric, and transdiaphragmatic pressure amplitudes (top panels) and areas (bottom panels) for balloon catheters and micro‐transducer catheter following cervical magnetic stimulation at increasing stimulation intensities. Data are mean ±SD and pooled from visits 1 and 2. Significant difference between catheters (*p < 0.05; **p < 0.01)

Bland–Altman plots for the agreement between the micro‐transducer and balloon catheters for P_es_, P_ga_, and P_di_ amplitudes and areas in response to cervical magnetic stimulation are shown in Figure 5. P_es_, P_ga_, and P_di_ amplitudes had biases of 3.8 (LOA −0.55 to 8.26), 4.2 (LOA −6.64 to 15.09), and 6.9 (LOA −0.61 to 14.27) cmH_2_O, respectively. Significant correlations between the catheters for P_es_ (*r* = 0.96), P_ga_ (*r* = 0.77), and P_di_ (*r* = 0.94) amplitudes were moderate to strong (*p* < 0.001). P_es_, P_ga_, and P_di_ pressure areas had biases of −0.08 (LOA −0.70 to 0.54), −0.03 (LOA −3.75 to 3.68), and −0.05 (LOA −1.22 to 1.11) cmH_2_O∙s, respectively. Significant correlations between the catheters for P_es_ (*r* = 0.94), P_ga_ (*r* = 0.84), and P_di_ (*r* = 0.91) were moderate to strong (*p* < 0.001).

Within‐ and between‐day reliability coefficients for P_es,_ P_ga_, and P_di_ amplitudes and areas in response to cervical magnetic stimulation at 100% of stimulator output for the micro‐transducer and balloon catheters are shown in Table 3. Within‐ and between‐day reliability for P_es_ and P_di_ amplitudes and areas were similar between the catheters. For the micro‐transducer compared to the balloon catheters, P_ga_ amplitudes, and areas had lower within‐day reliability and higher between‐day reliability.

**TABLE 3:** Experiment 2—*in vivo*: Within‐ and between day reliability of esophageal pressure (P_es_), gastric pressure (P_ga_), and transdiaphragmatic pressure (P_di_) amplitudes and areas for balloon catheters (BC) and micro‐transducer catheter (MC) following cervical magnetic stimulation at 100% of stimulator power output. Data are presented as means with 95% confidence intervals in parentheses

	P_es_		P_ga_		P_di_	
	BC	MC	BC	MC	BC	MC
*Within‐day (CV)*						
Amplitude (%)	8.7 (5.2 to 12.3)	10.7 (6.4 to 14.9)	6.7 (4.1 to 9.2)	12.9 (8.3 to 17.5)	6.2 (3.0 to 9.4)	6.1 (4.0 to 8.3)
Area (%)	14.5 (9.3 to 19.6)	12.8 (8.2 to 17.4)	14.9 (7.1 to 22.8)	23.4 (12.1 to 34.6)	9.6 (5.3 to 14.0)	8.6 (4.6 to 12.6)
*Between‐day (CV)*						
Amplitude (%)	10.7 (8.1 to 13.3)	10.9 (7.8 to 14.0)	20.7 (17.5 to 23.9)	17.8 (11.1 to 24.4)	9.8 (6.0 to 13.6)	11.3 (5.3 to 17.2)
Area (%)	15.0 (12.1 to 18.0)	16.0 (12.4 to 19.7)	30.6 (17.9 to 43.3)	26.4 (21.1 to 31.8)	13.0 (9.0 to 17.0)	18.5 (7.8 to 29.2)
*Between‐day (ICC)*						
Amplitude	0. 93 (0.69 to 0.99)	0.934 (0.70 to 0.99)	0.72 (−0.58 to 0.95)	0.60 (−1.54 to 0.92)	0.81 (−0.05 to 0.96)	0.82 (0.08 to 0.96)
Area	0. 94 (0.71 to 0.99)	0.903 (0.56 to 0.98)	0.68 (−0.92 to 0.93)	0.60 (−0.87 to 0.92)	0.79 (−0.12 to 0.96)	0.58 (−1.37 to 0.92)

Abbreviations: CV, coefficient of variation; ICC, intraclass correlation coefficient.

## 4 DISCUSSION

### 4.1 Main findings

This study is the first to provide a comprehensive analysis of a range of balloon and micro‐transducer catheter pressure measurement characteristics *in vitro* with a reference pressure following controlled pressurizations (Experiment 1) and *in vivo* following cervical magnetic stimulation (Experiment 2). The main findings were: (1) *in vitro* the micro‐transducer catheter showed closer agreement to the reference pressure amplitudes and areas than the balloon catheter; (2) *in vivo* the micro‐transducer catheter recorded higher pressure amplitudes and similar pressure areas than the balloon catheters; and (3) *in vivo* the micro‐transducer catheter displayed shorter pressure response times and half‐relaxation times, and greater maximal rates of changes in pressure than the balloon catheters.

### 4.2 Pressure amplitudes

*In vivo* the micro‐transducer catheter had higher pressure amplitudes compared to the balloon catheters. While no P_es_ agreement data following cervical magnetic stimulation have previously been reported, the values here are similar to those reported during quiet breathing (bias = −3.6 cmH_2_O, LOA −14.3 to 7 cmH_2_O) and demonstrate better agreement than those reported during sniff maneuvers (bias = −50.6 cmH_2_O, LOA −60.6 to −40.6 cmH_2_O) (Augusto et al., 2017). The presence of differences in pressure measurement is also consistent with previous work (Augusto et al., 2017; Beardsmore et al., 1982; Peters et al., 1998; Stell et al., 1999). The *in vivo* P_di_ results presented here with a bias of 6.9 (LOA −0.61 to 14.27) cmH_2_O are higher than those previously reported by Stell et al. with a bias of 2.1 (LOA −10.5 to 6.3) cmH_2_O. This difference is likely due to methodological and technical differences between the studies. For instance, Stell et al. placed micro‐transducer and balloon catheters simultaneously into their participants, thus exposing them to identical physiological conditions (i.e., excluding some of the within‐day variability potentially experienced during sequential catheter placements). The balloon catheters utilized by Stell et al. were from a different manufacturer, with a longer catheter (+24 cm) and balloons (+0.5 cm) and a different filling volumes for P_es_ (0.5 mL). These differences, respectively, may affect the dynamic compliance, whereas differences in balloon filling volumes affect pressure measurements (Cross et al., 2016; Milic‐Emili et al., 1964; Mojoli et al., 2015; Walterspacher et al., 2014). There are no published values of P_ga_ available against which to compare our results.

The *in vitro* results also demonstrated that the micro‐transducer catheter recorded higher pressure amplitudes than the balloon catheter and the pressures obtained were closer to the reference pressure. The differences in pressure amplitude between the catheters are likely due to the faster dynamic responses of the micro‐transducer catheter, allowing it to reach higher pressures more quickly than the balloon catheter, and thus more closely tracking rapid pressurization. *In vivo*, the within‐ and between‐day reliability coefficients for P_es_ and P_di_ amplitudes were similar between the catheters and to those reported previously for balloon catheters (Taylor & Romer, 2009; Tiller et al., 2017; Wüthrich et al., 2015). However, the within‐ and between‐day reliability coefficients for P_ga_ from the micro‐transducer catheter were higher than those of the balloon catheter and slightly higher than those reported previously for balloon catheters (Tiller et al., 2017). The differences may be explained by the greater sensitivity of the micro‐transducer catheter to pressure changes that occur readily within the stomach. The within‐day repeatability of pressure amplitudes and areas *in vitro* was high for both catheters, which suggests that when physiological factors are excluded, there are no inherent differences in the reliability of balloon and micro‐transducer catheters.

### 4.3 Pressure areas

The most common measurement of respiratory muscle strength is pressure amplitude (i.e., twitch pressures), however, the pressure area is also indicative of muscular work output (Bazzucchi et al., 2011; Carámbula et al., 2019; Celichowski et al., 2000). Areas have been reported for twitch tension (Lepers et al., 2000; Lewis et al., 2017) and twitch peak torque (Lepers et al., 2002) following electrical quadricep stimulations, but to the best of our knowledge have not been reported for the diaphragm following cervical magnetic stimulation. The pressure area envelope is “triangular” and pressure amplitude determines the perpendicular height of the triangle from base to apex, whereas the pressure response and relaxation rates control the slopes up and down from the apex. Thus, changes in pressure area are reflective predominantly of pressure amplitude, while also being influenced by differences in response and relaxation rates.

The micro‐transducer catheter demonstrated higher pressure amplitudes and sharper waveforms. Conversely, the balloon catheter displayed lower pressure amplitudes and blunter waveforms. Thus, despite the shape of the waveform recorded by the catheters being visibly different, the pressure areas are similar. This is evidenced *in vitro* by agreement values closer to zero and relative pressure area values that were closer to 100% for the micro‐transducer catheter. *In vivo* this is shown by the lack of main effect of catheter on pressure area results. However, the CV values for the within‐ and between‐day reliability indicate that pressure area measurements are less reliable than pressure amplitudes. Assessment of between‐day reliability using ICC indicates a higher degree of variability in P_ga_ and P_di_ amplitudes and areas as these values had wide CI, with some incorporating negative lower limits. While this indicates that the measures are unreliable, there is no significant evidence of differences in reliability between devices, or between pressure amplitudes and areas. Hence, these data indicate that pressure area could provide a measurement suitable for direct comparisons between micro‐transducer and balloon catheters.

### 4.4 Pressure responses, half‐relaxation times, and rates of pressure change

This is the first study to provide a comparative analysis of the pressure measurement characteristics of a micro‐transducer and balloon catheters following cervical magnetic stimulations. *In vivo*, the P_es_, P_ga_, and P_di_ responses of the micro‐transducer catheter had shorter latencies, 10–90% rise times, time to peak pressure and a greater MRPD than the balloon catheter in response to cervical magnetic stimulation. Furthermore, as pressures returned to baseline, the micro‐transducer catheter had shorter half‐relaxation times and greater maximal relaxation rates. No differences were observed in the time constant for P_es_, P_ga_, or P_di_. The larger variability of time constant values observed in P_ga_ (and thus P_di_) are due to the secondary peaks occurring in some gastric response curves. These alter the decay waveform from the standard exponential form, causing variability in the calculation of the time constant. Hence, caution is advised when collecting and analyzing time constant data. These response characteristic data show that the micro‐transducer catheter demonstrated “faster” responses to changes in pressures than balloon catheters. This does not imply that it performs better than the balloon catheter in measuring pressures *in vivo*. However, their faster responses do produce different waveforms in response to cervical magnetic stimulation, with the micro‐transducer catheter providing sharper and shorter response curves than the balloon catheters. The differences in catheter responses can be attributed to their unique designs, with the micro‐transducer having a greater inherent capacity for fast responses.

### 4.5 Methodological considerations

Experiment 1. Ideally any reference waveform used in in vitro respiratory testing should include waveforms with spectral content greater than 20 Hz. However, those presented in Experiment 1 were approximately 5 Hz and thus a deeper comparison of these data to assess the dynamic response characteristics of the catheters was not possible.

### 4.6 Clinical implications

Low P_di_ amplitudes (i.e., twitch pressures) in response to un‐potentiated cervical magnetic stimulation have been utilized for the identification of diaphragm weakness. Pressures below 20 cmH_2_O for bilateral phrenic nerve stimulation (such as that performed in this study) are potentially indicative of bilateral diaphragm weakness (ATS/ERS Taskforce, 2002). Pressures below 18 cmH_2_O correlate with observations of muscle weakness in some diseases (Steier et al., 2007), whereas those below 10 cmH_2_O in critically ill patients indicate acquired diaphragm weakness (Supinski & Callahan, 2013). Recently, Dubé and Dres (2016) produced algorithms for the suspicion and treatment of diaphragm dysfunction and proposed a twitch P_di_ <20 cmH_2_O (or <10 cmH_2_O for unilateral phrenic nerve stimulation) is indicative of bilateral diaphragm weakness. However, as these cut‐off values are based on respiratory pressures measured using balloon catheters, which based on our findings record lower P_di_. For example, the mean P_di_ twitch pressure for patients with severe stable COPD, measured using balloon catheters by Polkey et al., is 18.5 cmH_2_O (1996). If a micro‐transducer catheter was used, and the twitch P_di_ bias from our Experiment 2 (~6.9 cmH_2_O higher) factored in, the recorded value would have been closer to ~25.4 cmH_2_O indicating that diaphragm weakness is instead unlikely. Thus, applying the aforementioned cut‐off values measured using balloon catheters to those measured using a micro‐transducer catheter may lead to incorrect clinical assessments and diagnoses. This should therefore be considered if micro‐transducer catheters are used in the evaluation of diaphragm weakness, and it may be necessary to establish new normative and cut‐off values.

Alternatively, our results have demonstrated that a surrogate measurement for direct comparisons between micro‐transducer and balloon catheters may be pressure area, which corrects for differences in the pressure response shape between the catheters. If normative values and cut‐off values for pressure areas were ascertained, then these measurements would allow for comparisons between the catheters to be made. Given the presence of a main effect of catheter on P_di_, and the significant differences observed between catheters at 100% stimulation power, we would also expect significant differences between catheters when measuring potentiated twitch P_di_ (e.g., twitches delivered after a maximal volitional inspiratory maneuver). Thus, between catheter comparisons of diaphragm contractility test results should be interpreted with care. Response and relaxation rates (e.g., muscle shortening and relaxation rates) following cervical magnetic stimulation also provide valuable information pertaining to the mechanical properties of the diaphragm (ATS/ERS Taskforce, 2002, Laveneziana et al., 2019, Wilcox et al., 1988). The present study shows, however, that response and relaxation rates differ between the micro‐transducer and balloon catheters. Therefore, caution is warranted when comparing studies that have used different catheter systems to obtain these measurements.

## 5 CONCLUSION

This is the first study to provide a comparative analysis of the pressure measurement characteristics of micro‐transducer and balloon catheters in response to controlled pressurizations *in vitro* (Experiment 1) and cervical magnetic stimulations *in vivo* (Experiment 2). Under *in vivo* and *in vitro* conditions, the micro‐transducer catheter recorded higher pressure amplitudes, and under *in vivo* conditions, shorter response and relaxation rates and greater rates of changes in pressure compared to the balloon catheters. Accordingly, caution is warranted when comparing the results of studies that used different catheter systems to obtain these measurements. Furthermore, in a clinical setting caution is warranted if pressure amplitude measurements made with micro‐transducer catheters are compared to normative values derived from balloon catheters. However, this limitation may be mitigated if comparisons are made based on pressure area, which does not differ between micro‐transducer and balloon catheters.

## COMPETING INTERESTS

The authors declare no conflict of interest.

## AUTHOR CONTRIBUTIONS

W.M and D.E.M conceptualized and designed the experiments. W.M collected and analyzed the data. D.E.M, B.H contributed to data interpretation and statistical analysis. M.A.J and G.R.S contributed to revisions of intellectual content. All authors approved the final manuscript.
