# Corrigendum: Pressure measurement characteristics of a micro‐transducer and balloon catheters

**DOI:** 10.14814/phy2.15500

**Published:** 2022-11-02

The authors of the article by William MacAskill et al. (2021) noticed an error in the figure legends of their article. The correct figure legends are as follows:

Figure 2. Experiment 1 – in vitro: Ensemble average waveforms (each from 100 waves) from the micro‐transducer catheter (MC), balloon catheter (BC) and reference (RP) pressures in response to chamber pressures of 25, 50, 75 and 100 cmH2O with a constant pressurisation time of 0.2 s.

Figure 3. Experiment 2 – in vivo: Representative oesophageal, gastric and transdiaphragmatic pressure characteristics for the balloon catheters and micro‐transducer catheter following cervical magnetic stimulation at 100% of stimulator power output. Three repeated twitches from one participant are shown superimposed. Stimulation artefacts are marked with an arrow (↑).

Figure 4. Experiment 2 – in vivo: Oesophageal, gastric and transdiaphragmatic pressure amplitudes (top panels) and areas (bottom panels) for balloon catheters and micro‐transducer catheter following cervical magnetic stimulation at increasing stimulation intensities. Data are mean ± SD and pooled from visits 1 and 2. Significant difference between catheters (*P < 0.05; **P < 0.01).

Figure 5. Bland–Altman plots of oesophageal, gastric and transdiaphragmatic pressure amplitudes (top panels) and areas (bottom panels) between balloon catheters (BC) and micro‐transducer catheter (MC) following cervical magnetic stimulation at increasing stimulation intensities. Bias is represented by the solid line and the limits of agreement by the dotted lines (± 1.96 SD). Each participant has one datapoint per stimulation power and each datapoint was calculated as the mean value from visits 1 and 2.
